# Distinct psychological profiles and responsiveness to a brief intervention in workers with high versus low intensity emotional labor: an observational study

**DOI:** 10.1371/journal.pone.0345553

**Published:** 2026-05-06

**Authors:** Dasom Lee, Nahyun Ha, Changyoung Oh, Ul Soon Lee, YoonJi Irene Lee, Do-Hyung Kang, Soo-Hee Choi

**Affiliations:** 1 Department of Psychiatry, Seoul National University Hospital, Seoul, Republic of Korea; 2 Department of Brain Training, Global Cyber University, Cheonan, Republic of Korea; 3 Kang Do-Hyung Psychiatry Clinic, Seoul, Republic of Korea; 4 Department of Psychiatry, Seoul National University College of Medicine and Institute of Human Behavioral Medicine, SNU-MRC, Seoul, Republic of Korea; University of Toronto, CANADA

## Abstract

Emotional labor refers to the process by which employees regulate and manage their emotions as part of their job requirements. This observational study examined distinct psychological profiles and differential responsiveness to a brief 35-minute mind-body training (MBT) among 753 emotional labor workers with high versus low emotional labor intensity. Participants were categorized into high-risk and low-risk groups based on their emotional labor intensity. Psychological measures included positive and negative affect (Positive and Negative Affect Schedule), depressed mood (Center for Epidemiologic Studies Depression Scale), and quality of life (World Health Organization Quality of Life-Brief Version). Resting heart rate was obtained from a limited sample (*n* = 29) for exploratory analysis. At baseline, among emotional labor subscales, *emotional disharmony and hurt* was associated with increased depressed mood and decreased quality of life in both groups (*p* < .001), while *lack of a supportive and protective system in the organization* positively correlated with depressed mood in the high-risk group only (*p* = .040). After the MBT, the high-risk group showed greater decrease in negative affect compared to the low-risk group (*p* < .001). Exploratory analysis showed heart rate decreased significantly regardless of group (*p* = .039), with greater reductions in employees reporting higher baseline *emotional demand and regulation* (*r* = .464, *p* = .01). Our results suggest that high-risk workers, particularly those lacking organizational support, may exhibit greater psychological vulnerabilities but also greater responsiveness to workplace intervention, providing preliminary evidence for considering emotional labor intensity when designing workplace interventions.

## Introduction

Emotional labor refers to the process by which employees are required to regulate and manage their emotions as part of their job, in accordance with organizationally prescribed rules and guidelines [1]. Employees typically adopt emotional labor strategies which are generally categorized into surface acting (i.e., faking or suppressing true feelings) and deep acting (i.e., attempting to genuinely feel the required emotion) [1]. Research has demonstrated that consistently suppressing one’s true emotions to display organizationally desired ones can lead to increased physiological and psychological stress responses [1–5]. Furthermore, a comprehensive report from an occupational safety and health agency revealed that employees engaged in high-intensity emotional labor were 2.44 to 3 times more likely to screen positive for depression compared to those performing low-intensity emotional labor [6]. The study also found that these workers not only experienced a 2.86 to 3.07 times higher prevalence of clinical insomnia, but also demonstrated a 2.02 times greater likelihood of reporting suicidal ideation. These adverse effects and its consequent impact on productivity have prompted researchers to focus on categorizing or subtyping emotional labor workers and identifying vulnerable subgroups within this population [6–9]. In a previous study that categorized male firefighters into high-risk and normal groups based on emotional labor intensity [9], the high-risk group was associated with higher levels of depressed mood and insomnia, as well as lower levels of post-sleep fatigue recovery and resilience. These findings highlight the severe psychological consequences of high-intensity emotional labor, emphasizing the urgency of identifying and supporting these vulnerable groups. To effectively identify at-risk employees and develop appropriate support measures, more studies should explore which aspects of emotional labor are particularly detrimental to psychological well-being and thus should be targeted for intervention. The Job Demands-Resources (JD-R) model may provide a theoretical framework for understanding and addressing these vulnerabilities [10,11]. According to the JD-R model’s health impairment process, chronic imbalance between high job demands (such as emotional labor) and insufficient resources leads to energy depletion, burnout, and serious health deterioration [12]. Therefore, it is necessary to comprehensively assess job demand and resource factors related to emotional labor and examine their effects on psychological well-being.

Identifying at-risk employees is particularly important given a recent report that workers’ emotional labor strategies, especially maladaptive ones that are more detrimental to psychological well-being, tend to remain stable over time [8]. Specifically, the study identified distinct profiles of emotional labor based on employees’ use of different strategies, with profiles characterized by high levels of surface acting exhibiting the lowest job satisfaction and the highest emotional exhaustion and turnover intentions. A within-person stability analysis revealed that 95.4% of this subgroup remained in the same profile after three months, indicating that this group is particularly resistant to transitioning into more adaptive coping styles [8]. The persistence of the use of emotional labor strategy highlights the need for organizational-level screening and targeted interventions to address the enduring negative effects of emotional labor in this high-risk group. To effectively implement these measures, it is crucial to identify the specific aspects of emotional labor that have significant impact on psychological well-being. This targeted approach would enable organizations to develop more precise and effective interventions.

Building upon the need for proactive organizational measures, it is essential to explore specific interventions that can be effectively integrated into the work environments. Among these, meditation stands out as a promising approach that can be implemented in a naturalistic and cost-effective manner. The efficacy of meditation has been well-established through previous research, with studies showing that meditation can alleviate a variety of medical and psychiatric conditions, including cardiovascular disease and depression [13–20]. One of the key mechanisms is that meditation enhances autonomic regulation by promoting parasympathetic over sympathetic activity, thereby facilitating emotional and physiological relaxation [13,14,21,22]. Given that persistent emotional suppression and control during emotional labor can result in prolonged autonomic arousal and consequent cardiovascular and immune dysfunction [1,3,4], interventions targeting autonomic regulation become particularly relevant. For example, workplace-based mindfulness programs have been reported to reduce cortisol production and sympathetic nervous system reactivity, as well as improve autonomic balance and immune function [23]. Besides physiological relaxation, meditation is known to improve positive and negative affect [16]. According to Affective Events Theory (AET) [24], employee attitudes and behaviors are not merely products of stable work environments (e.g., work intensity) but are primarily driven by specific daily work events that trigger internal emotional reactions. Indeed, a study of call center workers revealed that affect mediated the relationship between work characteristics and employee health and job satisfaction [25]. Specifically, work overload induced negative emotions that led to health problems, whereas positive work characteristics such as autonomy, supervisor support, and welfare enhanced job satisfaction through positive affect. These findings suggest that managing employees’ daily affective experiences may be critical for workplace well-being. From this perspective, meditation-based interventions become particularly relevant. In addition, from the JD-R perspective, job resources including institutional and social support become increasingly critical as job demands increase [12,26,27]. This theoretical framework suggests that the protective effects of resources are amplified under high-demand conditions, helping to buffer against work-related strain. Applied to meditation interventions, this may imply that the effects of meditation may differ depending on emotional labor intensity. However, few studies have investigated whether employees with different levels of emotional labor intensity would exhibit differential responsiveness after meditation interventions. Taken together, while research on emotional labor and its intervention strategies has grown substantially, important empirical questions remain unexplored. First, comparative analyses of how specific dimensions of emotional labor differentially affect psychological outcomes in high-risk versus low-risk workers are limited. Second, although vulnerable subgroups have been identified [8], their distinct psychological profiles require further characterization. Third, despite meditation showing promise as a workplace intervention, whether psychological changes after meditation vary across emotional labor risk group remains largely unexplored. As emotional labor becomes a defining feature of more occupations, developing evidence-based, tailored intervention strategies has become critical for protecting worker well-being and organizational sustainability. Addressing these needs, the present study examined how specific emotional labor characteristics relate differently to depressed mood and quality of life in high- vs. low-risk emotional labor groups, and compared their responsiveness to a meditation intervention.

Previous studies have demonstrated that an 8-week mind-body training (MBT) program incorporating movement-based meditation effectively reduces stress and enhances emotional intelligence, resilience, and coping abilities [28,29]. In addition, a 4-week MBT was found to improve functional connectivity within the default mode network [30]. Although it was once believed that intensive and prolonged (months to years) meditation is required for beneficial effects, recent studies showed that brief meditation is sufficient to induce mental, physical, and neuronal changes [31,32]. For example, daily meditation (20 min for 3–5 days) was effective in improving autonomic regulation and cognitive functions including attention, working memory, and executive control [33,34], and enhancing physiological relaxation as evidenced by decreased heart rate [35]. Given its accessibility, cost-effectiveness, and demonstrated benefits, meditation-based interventions could serve as a valuable tool in mitigating the adverse effects of emotional labor. Based on these findings, the present study adapted the previously validated 4-week and 8-week MBT programs [28–30] into a condensed 35-minute single session format that can easily be integrated into the work setting, and applied the program to high- and low-risk emotional labor groups. In particular, we aimed to identify how the subfactors of emotional labor relate differently to depressed mood and quality of life in each group. Given the accumulating evidence on the benefits of MBT [28–30], the present study focused on comparing immediate responsiveness to the MBT between the two groups. In addition, we conducted exploratory physiological analyses to examine which dimensions of emotional labor may be associated with autonomic changes following the MBT.

## Materials and methods

### Study design

The study protocol was approved by the Institutional Review Board of Seoul National University Hospital, Republic of Korea (IRB No. 1706-027-857). The study was conducted in accordance with the Declaration of Helsinki. Written informed consent was obtained from all participants. This observational study employed a prospective naturalistic intervention design without a control group. We conducted a single MBT session at participants’ worksites and assessed outcomes at baseline and immediately post-intervention. Participants were stratified into high-risk and low-risk groups based on emotional labor intensity to examine differential responsiveness to the intervention. Psychological and physiological measures were collected at both time points, while physiological data were obtained from a subset of participants. Follow-up data were additionally collected approximately 3 months after the intervention; however, these data were not reported in the present study. Details of the follow-up data collection procedures and the corresponding datasets are provided in the Supporting Information.

### Participants

Participants were recruited from August 18, 2017 to December 7, 2017. Eligible participants were 20–50-year-old emotional laborers whose jobs required face-to-face or voice-to-voice interaction with customers. This age range was selected to reflect the core working-age population most representative of emotional labor occupations in South Korea, while minimizing potential confounds from age-related health conditions. Exclusion criteria were participants with difficulty in understanding or responding to self-administered questionnaires, or serious medical, orthopedic, or neurological disorders. Participants were employees from companies (e.g., hospitals, call-centers, and civil affairs centers) that volunteered to participate in the MBT program. Companies were recruited through online advertisements and direct email invitations from the research team. As the study design was observational, participants were not assigned to an intervention or a control group. Employees at participating companies were informed about the program through internal announcements, and provided informed consent for voluntary participation. A total of 35 sessions were conducted across 21 worksites, with larger organizations divided into multiple sessions to accommodate all interested employees. In total, 808 individuals participated in the study. Among these participants, 779 (96.4%) completed pre-training questionnaires and training, and 753 (93.2%) completed both pre- and post-training questionnaires (S1 Fig). For physiological data collection, a limited number of participants (n = 36) completed pre-training physiological recordings, and 35 completed post-training recordings.

### Categorizing emotional labor groups

The level of emotional labor was assessed using the Korean Emotional Labor Scale (K-ELS) [36]. The K-ELS was validated through a nationwide validation study conducted by Korean Occupational Safety & Health Agency using a random sample of 1,042 Korean employees [37]. It has been widely used in previous studies, including those examining employees’ suicidal ideation, burnout, sleep disturbance, work-related symptoms of posttraumatic stress disorder, and the effectiveness of stress reduction programs [38–44]. The scale comprises five subscales: five items of *emotional demand and regulation (EL1, e.g., “I consciously make an effort not to express negative emotions to customers.”),* three items of *overload and conflict in customer service (EL2, e.g., “I have to deal with aggressive or demanding customers.”),* six items of *emotional disharmony and hurt (EL3, e.g., “I feel hurt when I have to hide and suppress my emotions from customers.”),* three items of *organizational surveillance and monitoring (EL4, e.g., “I am monitored (e.g., through CCTV) to ensure I interact with customers according to workplace requirements.”),* and seven items of *lack of a supportive and protective system in the organization (EL5, e.g., “There are formal systems and procedures in place at work to help resolve and support issues that arise during customer interactions.”,* reverse-scored*)*. It consists of a total of 24 items rated on a 4-point Likert scale, with higher scores indicating greater emotional labor at work. As in previous studies [6,9], the sum of the first three subscales was defined as the intensity of emotional labor. Each subscale and the sum scores were converted to a score out of 100. In this study, the Cronbach’s alpha for the five subscales ranged from 0.669 to 0.878, demonstrating acceptable internal consistency.

A national research report of Korea Occupational Safety and Health Agency used receiver operating characteristic (ROC) curve analysis to determine the cut-off values for emotional labor scores in classifying depression [6]. In this analysis, the Patient Health Questionnaire-9 (PHQ-9) was used as the criterion standard for depression [45]. ROC curves were constructed to evaluate how well emotional labor intensity scores could predict PHQ-9-defined depression. The analysis identified optimal cut-off points—60.36 for males and 66.67 for females—at which the predictive performance was maximized. Individuals scoring above these thresholds were classified as being at high-risk for depression. As in a previous study [9], we adopted these cut-off values in our study to categorize employees into the high-risk (*n* = 332) and the low-risk (*n* = 391) emotional labor groups. Table 1 presents detailed demographic and work-related features of the two groups.

**Table 1:** Baseline demographic, work-related, and outcome measures of high-risk vs. low-risk emotional labor groups.

Variable	Group		Statistics
	High-risk (*n* = 332)	Low-risk (*n* = 391)	
	N (%) or mean (SD)	N (%) or mean (SD)	*p*
**Demographic features**			
Age, year	36.87 (8.22)	36.10 (8.58)	.215^a^
Female, %	305 (91.9)	347 (88.7)	.170^b^
Married, %	181 (54.5)	205 (52.4)	.780^b^
University degree, %	246 (74.1)	307 (78.5)	.250^b^
**Work-related features**			
Length of employment, months	66.22 (73.88)	70.01 (77.73)	.509^a^
Permanent employment, %	214 (64.5)	247 (63.2)	.373^b^
Weekly working hours	42.56 (6.19)	41.73 (5.61)	.090^a^
Workplace, %			**<.001** ^b^
Hospital	117 (35.2)	143 (36.6)	
Civil affairs centers	59 (17.8)	120 (30.7)	
Call centers	156 (47.0)	128 (32.7)	
Emotional labor score			
EL1	82.33 (13.11)	64.93 (13.26)	**<.001** ^a^
EL2	82.86 (14.75)	49.15 (18.00)	**<.001** ^a^
EL3	72.19 (15.96)	42.86 (15.44)	**<.001** ^a^
EL4	57.58 (21.37)	36.54 (18.12)	**<.001** ^a^
EL5	49.25 (15.46)	49.03 (13.72)	.840^a^
**Psychological measures**			
Positive affect	20.99 (6.74)	22.59 (7.26)	**.002** ^a^
Negative affect	20.22 (7.62)	17.49 (6.57)	**<.001** ^a^
Depressed mood	19.52 (9.12)	15.57 (8.30)	**<.001** ^a^
Quality of life			
Physical	12.84 (2.33)	13.94 (2.21)	**<.001** ^a^
Psychological	11.36 (2.60)	12.48 (2.49)	**<.001** ^a^
Social	12.89 (2.51)	13.36 (2.33)	**.010** ^a^
Environment	11.80 (2.52)	12.54 (2.23)	**<.001** ^a^

SD, standard deviation; EL1, emotional demand and regulation; EL2, overload and conflict in customer service; EL3, emotional disharmony and hurt; EL4, organizational surveillance and monitoring; EL5, lack of a supportive and protective system in the organization.

a, b indicates p values derived from independent samples t-tests and chi-square tests, respectively.

### Occupational stress management program

The program utilized in the present study was a brief version of the 4-week and 8-week MBT programs that have been reported to yield beneficial effects on various psychological factors, including stress response, coping strategies, emotional intelligence, resilience, and negative emotions [28,29]. These programs have been shown to influence functional connectivity within the default mode network, a brain network associated with self-referential processing and emotional regulation [30]. To adapt these programs for workplace settings, the content was condensed into a single 35-minute session. This practical adaptation is supported by a growing body of literature demonstrating that brief single meditation sessions (< 20 minutes) are effective in improving state mindfulness, cognitive function, anxiety, and mood [46–49]. Consistent with the original 4-week and 8-week MBT protocols, this brief program was developed and conducted in Korean.

The MBT program was delivered to employees in a group session format during allocated time for staff stress management training. The session was guided by a qualified psychiatrist and meditation expert with relevant coaching experience. The psychiatrist began the session with a brief 20-minute lecture using audiovisual materials to educate participants about emotional labor and its effects on the body. Following the lecture, the 35-minute MBT program was conducted in accordance with the protocol. The program consisted of yoga-like stretching exercises and mindfulness meditation with breathing techniques.

The first 15 minutes were dedicated to stretching exercises specifically targeting areas commonly affected by workplace stress, focusing on relaxing the neck, shoulders, and upper back. Exercises included simple stretches such as raising interlaced hands overhead and leaning to each side, stretching the chest by extending arms behind the back, gently moving the head up and down to stretch the neck, and lifting shoulders up toward the ears, then letting them drop down. The instructor guided participants to consciously focus on the physical sensations of muscles contracting and relaxing in different areas of the body during each stretching movement [50]. This attentional focus on bodily sensations during stretching is thought to engage neural networks associated with interoceptive and emotional processing, which may contribute to improvements in attentional functioning and stress reduction in meditation practitioners [51].

The subsequent 20-minute mindfulness session was guided by a meditation expert. Participants engaged in diaphragmatic breathing while maintaining present-moment awareness of bodily sensations and emotional states. They were instructed to inhale deeply through the nose as their abdomen expanded and exhale slowly through the mouth as it contracted, focusing attention on these abdominal movements. The guided practice incorporated non-judgmental observation of negative emotions, teaching participants to acknowledge emotions without suppression. This approach was designed to facilitate emotional release by promoting relaxation and mindfulness, allowing individuals to observe thoughts and emotions from a detached perspective, reduce the intensity of negative affect, and foster a sense of letting go [50]. The diaphragmatic breathing component promotes physiological relaxation by reducing muscle tension and activating the parasympathetic nervous system, which helps restore autonomic balance and alleviate stress-related arousal [50,52]. Mindfulness training has been reported to strengthen executive control by training participants to maintain attention on present-moment bodily sensations and emotional states [53–55]. Enhanced attentional control is considered to support the capacity to observe emotions non-judgmentally and reduces habitual reactivity to stressors, which may facilitate adaptive emotion regulation processes including cognitive reappraisal [56].

### Questionnaires

Participants completed the self-administered questionnaires immediately before and after MBT. Positive and negative affect were measured at both time points, while depressed mood and quality of life were measured only at baseline.

*Positive and Negative Affect*. Positive and negative affect were measured using the Korean version of the Positive Affect and Negative Affect Schedule (PANAS), which consists of 10 positive (interested, alert, attentive, excited, enthusiastic, inspired, proud, determined, strong, and active) and 10 negative (distressed, upset, guilty, ashamed, hostile, irritable, nervous, jittery, scared, and afraid) affect descriptors [57]. Each item was scored on a 5-point Likert-type scale, with higher scores indicating higher levels of affect. The Cronbach’s alpha for the two subscales in this study ranged from 0.890 to 0.894.

*Depressed mood.* Depressed mood was evaluated using the Korean version of the Center for Epidemiologic Studies Depression (CES-D) scale [58]. The CES-D consists of 20 items that measure the number and duration of symptoms experienced over the preceding 2 weeks. For each symptom, responses were scored on a 4-point Likert scale. The Cronbach’s alpha in this study was 0.911.

*Quality of life.* Quality of life was measured using the Korean version of World Health Organization Quality of Life-Brief Version (WHO-QOL) [59]. This scale consists of 26 items, divided into two general categories (overall quality of life and general health) and four domains (physical, psychological, social, and environmental). In the present study, the scores from the four domains were used for analysis. Higher scores indicated better quality of life in each domain. The Cronbach’s alpha for the four domains in this study ranged from 0.723 to 0.813.

### Physiological data

Previous research examining meditation effects has commonly employed heart rate and high frequency heart rate variability as measures of parasympathetic nervous system activity [60]. Given that MBT incorporates relaxation breathing and mindfulness, the present study measured these same physiological indicators along with low frequency heart rate variability to examine associated changes. For participants who underwent physiological measurements, 5-min resting-state heart rate was recorded after the questionnaires had been completed [61]. The participants wore a blood volume pulse sensor (MindMedia BVP sensor, Herten, Netherlands) on their right index finger. Data were acquired using the Nexus10-Mark2 device at a sampling rate of 256 Hz and analyzed using BioTrace+ software (MindMedia Lab). Physiological data were automatically generated, including heart rate (beats/min) and heart rate variability in the frequency domain, specifically low frequency (LF) and high frequency (HF) components. LF (0.04 to 0.15 Hz) reflects sympathetic and parasympathetic nerve activities [62], while HF (0.15 to 0.4 Hz) represents activation of the parasympathetic system or vagus nerve and is generally used as a physiological marker of relaxation in meditation research [60]. In this study, normalized values of LF and HF (nLF and nHF) were used.

### Statistical analysis

For the psychological measures analysis, questionnaire data from 723 out of 753 participants were included. Participants were excluded due to missing emotional labor scores (9 participants) and missing information on religion (21 participants), which was used as a covariate in subsequent analyses. For the physiological data analysis, 29 out of 35 participants were included. Six participants were excluded due to missing baseline emotional labor scores. To analyze the baseline differences between the high-risk and the low-risk group, we conducted independent samples t-tests and chi-square tests on demographic and work-related features, as well as psychological (positive/negative affect, depressed mood, quality of life) outcome measures. Prior to these analyses, the Shapiro-Wilk test indicated that all psychological variables met the normality assumption.

Next, we investigated how emotional labor subscales were related to baseline depressed mood and quality of life using stepwise multiple linear regression analyses. Separate models were constructed for each group, with depressed mood or each of the four domains of quality of life serving as the dependent variable in each model. The 5 subscales of emotional labor were entered as independent variables in each model to determine which subscales significantly explain depressed mood or quality of life. Standardized regression coefficients (*β*) were reported to assess the relative importance of each independent variable, and the overall model fit was evaluated using the adjusted *R*^2^ value. Assumptions of multiple regression, such as linearity, normality of residuals, homoscedasticity, and absence of multicollinearity, were checked to ensure the validity of the results. Multicollinearity was evaluated using Variance Inflation Factor (VIF) scores. All independent variables exhibited VIF values below 1.113, confirming the absence of multicollinearity.

Changes following MBT were examined on positive and negative affect. We performed 2 × 2 repeated measures analysis of covariance (rmANCOVA) with Group (high-risk vs. low-risk) and Time (pre- vs. post-training) as independent variables. To control for the potential influence of participants’ familiarity with meditation, participants’ current meditation experience and religious affiliation were included as covariates.

For physiological outcomes, group comparisons were not reported due to insufficient statistical power (high-risk: n = 10; low-risk: n = 19). Instead, exploratory analyses were conducted with groups combined (n = 29) in order to 1) examine overall physiological changes following MBT using rmANCOVA, and 2) identify baseline emotional labor characteristics associated with physiological responsiveness using Pearson correlation analyses.

P-values < .05 were considered statistically significant, without correction for multiple comparisons. Effect sizes for each dependent variable were reported as the partial eta-squared (*η²p*), where applicable. All analyses were performed using SPSS software (version 25.0; IBM Corp., Armonk, NY, USA).

## Results

### Baseline group differences

First, the baseline demographic and work-related features were compared between the high- vs. low-risk groups (Table 1). Comparisons of demographic characteristics revealed no significant group differences in age, sex, marital status, and education level (all, *p* > .05). Comparisons of work-related features showed that the proportion of workplaces differed between the two groups (*p* < .001). The high-risk group had more call center workers (47.0%) and fewer civil affairs center workers (17.8%), while the proportion of hospital workers was similar in both groups. However, there was no significant group difference in the length of employment (*p* = .509), the proportion of permanent and temporary workers (*p* = .373), and the weekly working hours (p = .090). Most of emotional labor subscales were significantly higher in the high-risk group (all, *p* < .001), except that EL5 did not differ between the two groups (*p* = .840).

Next, the baseline psychological measures were compared between the two groups (Table 1). The high-risk group had significantly higher levels of negative affect (*p* < .001) and depressed mood (*p* < .001) with lower levels of positive affect (*p* = .002) and worse quality of life (all, *p* < .010).

### The relationships between emotional labor and psychological well-being

**Fig 1:**
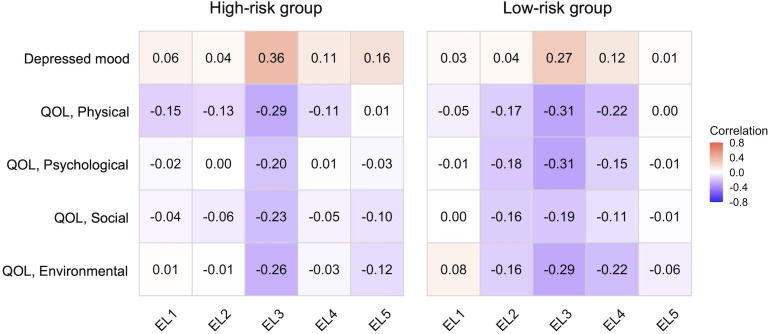
The correlation heatmap of the association of emotional labor subscales with depressed mood and quality of life. EL1: emotional demand and regulation, EL2: overload and conflict in customer service, EL3: emotional disharmony and hurt, EL4: organizational surveillance and monitoring, EL5: lack of a supportive and protective system in the organization, QOL: quality of life.

Fig 1 demonstrates a correlation heatmap of the association of emotional labor subscales with depressed mood and quality of life in the two groups. Multiple regression analyses on depressed mood revealed that, in both groups, EL3 was positively associated with depressed mood (Table 2). Notably, EL5 showed a positive association with depressed mood only in the high-risk group (*β* = 0.11, *p* = .040). These findings suggest that the factors accounting for depressed mood may differ between the two groups, with *lack of a supportive and protective system in the organization* playing a more significant role in the high-risk group.

**Table 2:** Stepwise multiple linear regression analysis examining associations between emotional labor subscales and psychological well-being in the high-risk and low-risk group.

	High-risk group (*n* = 332)						
	Independent variables					Statistics	
Dependent variables	EL1	EL2	EL3	EL4	EL5	*R* ^2^	*F*
Depressed mood	0.004 (0.07)	−0.05 (−0.87)	0.32 (6.11**)	−0.06 (−1.08)	0.11 (2.06*)	.123	23.20**
QOL, Physical	−0.07 (−1.25)	−0.03 (−0.58)	−0.24 (−4.35**)	−0.01 (−0.22)	0.02 (0.31)	.053	18.88**
QOL, Psychological	−0.01 (−0.16)	0.07 (1.15)	−0.20 (−3.70**)	0.09 (1.57)	−0.04 (−0.64)	.038	13.71**
QOL, Social	0.01 (0.10)	0.02 (0.39)	−0.19 (−3.43**)	0.09 (1.52)	−0.04 (−0.68)	.032	11.76*
QOL, Environmental	0.04 (0.75)	0.08 (1.37)	−0.23 (−4.32**)	0.02 (0.33)	−0.08 (−1.41)	.051	18.69**
	**Low−risk group (*****n*** **= 391)**						
	**Independent variables**					**Statistics**	
**Dependent variables**	**EL1**	**EL2**	**EL3**	**EL4**	**EL5**	***R*** ^**2**^	***F***
Depressed mood	0.01 (0.27)	−0.04 (−0.65)	0.25 (4.87**)	0.03 (0.61)	0.04 (0.75)	.058	23.74**
QOL, Physical	−0.003 (−0.06)	−0.06 (−1.12)	−0.25 (−4.96**)	−0.11 (−2.15*)	0.02 (0.35)	.089	19.72**
QOL, Psychological	0.06 (1.31)	−0.17 (−3.38*)	−0.26 (−5.16**)	−0.06 (−1.15)	0.02 (0.42)	.117	26.40**
QOL, Social	0.07 (1.33)	−0.12 (−2.32*)	−0.18 (−3.40*)	−0.01 (−0.18)	−0.05 (−0.92)	.055	11.90**
QOL, Environmental	0.13 (2.59^*^)	−0.03 (−0.49)	−0.25 (−4.86**)	−0.15 (−2.93*)	−0.05 (−0.97)	.108	16.54**

Data are presented as standardized beta coefficients (t value).

** *p* < 0.001, * *p* < 0.05.

EL1: emotional demand and regulation, EL2: overload and conflict in customer service, EL3: emotional disharmony and hurt, EL4: organizational surveillance and monitoring, EL5: lack of a supportive and protective system in the organization, QOL: quality of life.

Multiple regression analyses examining the relationship between emotional labor factors and each domain of quality of life revealed distinct patterns in the two groups (Table 2). In both groups, EL3 showed negative relationships with all quality of life domains. In the low-risk group, EL4 was negatively associated with the physical and environmental domains. Notably, in the low-risk group, EL2 was negatively associated with psychological and social domains, suggesting a more pronounced role of work-related stress in this group. Interestingly, EL1 was positively associated with environmental quality of life in the low-risk group, indicating that some aspects of emotional management might be linked to better perceived quality of life in the low-risk group, but not in the high-risk group.

### Changes following the MBT

Table 3 summarizes the changes in psychological and physiological outcome measures in each group. There was a significant main effect of Time (pre- vs. post-training) on positive affect (*F*[1,719] = 343.22, *p* < .001, *η²*_*p*_ = .32), indicating that positive affect increased following MBT (pre-training: 21.78; post-training: 28.68). The main effect of Group revealed that, regardless of Time, the level of positive affect was greater in the low-risk group (*F*[1,719] = 9.25, *p* = .002, *η²*_*p*_ = .013). There was no significant interaction of Time x Group (*F*[1,719] = 1.01, *p* = .31, *η²*_*p*_ = .001).

**Table 3:** Changes following the MBT.

Self-reported questionnaire							
	High-risk (*n* = 332)		Low-risk (*n* = 391)		F		
	Pre	Post	Pre	Post	Time	Group	Interaction
**Positive affect**	20.91 (0.39)	28.08 (0.43)	22.66 (0.36)	29.27 (0.40)	**343.22** ^**^	**9.25** ^*^	1.01
**Negative affect**	20.24 (0.39)	13.31 (0.26)	17.47 (0.36)	12.22 (0.24)	**357.06** ^**^	**25.30** ^**^	**12.60** ^**^

Data are given as adjusted mean (standard error).

* *p* < .05, ** *p* < .001.

For negative affect, the results showed significant interaction of Time x Group (*F*[1,719] = 12.60, *p* < .001; *η²*_*p*_ = .017) as well as significant main effect of Time (*F*[1,719] = 357.06, *p* < .001, *η²*_*p*_ = .33) and Group (*F*[1,719] = 25.30, *p* < .001, *η²*_*p*_ = .034). Subsequent independent t-test revealed a greater decrease in negative affect seen in the high-risk compared to the low-risk group (*t* = 3.37, *p* = .001). However, when controlling for job type and baseline severi*t*y, changes in negative affect were comparable between the two groups (*F*[1,719] =.179, *p* = .673).

### Physiological variables

There was a significant main effect of Time (*F*[1,25] = 4.73, *p* = .039, *η²*_*p*_ = .159) on heart rate; the heart rate decreased from 75.35 ± 9.72 to 73.17 ± 9.83 right after the training. Regarding nLF and nHF, there were no significant main or interaction effects (all, *p* > .05).

Further investigation into which dimensions of emotional labor were associated with physiological responsiveness revealed that a decrease in heart rate was positively correlated with baseline EL1 score (*r* = .464, *p* = .010). This indicates that employees who exert greater effort to regulate emotions at work demonstrated greater physiological relaxation after the training. Other subscales of emotional labor did not correlate with changes in heart rate, nLF, or nHF (all, *p* > .05).

## Discussion

This observational study demonstrated that the relationships between emotional labor and psychological outcomes differed between high-risk and low-risk emotional labor groups. In both groups, *emotional disharmony and hurt* caused by emotional labor was associated with exacerbated depressed mood and reduced quality of life. However, *lack of a supportive and protective system in the organization* was significantly associated with depressed mood only in the high-risk group, suggesting a need for organizational-level interventions to effectively support the psychological well-being of high-risk emotional laborers. Regarding the program, both groups showed increases in positive affect and decreases in negative affect after MBT, with a greater reduction in negative affect observed in the high-risk group.

*Emotional disharmony and hurt*, considered to have the greatest impact among emotional labor subscales, has been associated with various negative outcomes including turnover intention and suicidal ideation in previous studies [42,44,63,64]. While emotional labor itself may not be inherently negative, the failure to regulate emotions effectively could be detrimental to psychological well-being. Thus, workplace programs focusing on emotional regulation skills could be beneficial [65–67]. Notably, depressed mood in the high-risk group was also associated with *lack of a supportive and protective system in the organization* in the present study, suggesting that the positive relationship between organizational support/protection and depressed mood may be more pronounced for workers experiencing high-intensity emotional labor. This aligns with contemporary occupational stress theories including the JD-R model, which suggests that job resources including institutional and social support become increasingly critical as job demands increase [12,26,27]. In high-stress occupational contexts, the protective effect of these resources is considerably enhanced, significantly buffering against work-related psychological strain [12,26,27]. Accordingly, high-risk workers may rely more on these support systems to mitigate psychological strain. Consistent with this, the high-risk group in this study showed a greater decrease in negative affect following the MBT compared to the low-risk group. This differential response in negative affect could indicate that high-risk group may be more reliant on and responsive to organizational support programs.

However, the greater reduction in negative affect observed in the high-risk group was no longer significant after controlling for job type and baseline severity. The high-risk group in our study is characterized by a higher proportion of occupations with low job autonomy (e.g., call-center) and greater baseline psychological burden, indicating that risk grouping reflects a cluster of workers with both demanding occupational contexts and heightened vulnerability. Accordingly, group differences in changes following the MBT should be interpreted in light of these occupational and baseline factors, rather than attributed to risk grouping alone. Future studies with samples balanced for job type and baseline severity are needed to clarify whether emotional labor intensity has additional explanatory power beyond these characteristics. Changes following the MBT by job type are summarized in S4 Table. Taken together, these findings underscore the need to consider emotional labor intensity in conjunction with occupational and baseline vulnerabilities when designing workplace interventions, and to develop tailored strategies that provide organizational support and address the heightened negative affect observed in high-risk workers.

In the low-risk emotional labor group, quality of life scores in the environmental domain increased with higher levels of *emotional demand and regulation*, while this was not observed in the high-risk group. This suggests that in the low-risk group, higher scores on *emotional demand and regulation* reflecting individual efforts in emotion regulation and the diversity of emotional expression during customer interactions may be related to a better perception of their environmental well-being. Prior research has similarly noted that emotional labor does not uniformly exert negative influences; for example, some studies have found positive associations between emotional labor levels and job satisfaction [68]. Ashforth and Humphrey (1993) proposed that displaying emotions appropriate to one’s job role can potentially make customer interactions more structured and predictable, helping workers avoid embarrassing interpersonal problems [69]. However, in the present study, such associations were not observed in the high-risk group. It is possible that, under conditions of high emotional labor intensity, organizational support and protection may play a more important role in sustaining workers’ mental well-being. In addition, *overload and conflict in customer service* and *organizational surveillance and monitoring* were negatively associated with quality of life only in the low-risk group. This discrepancy may be due to a ceiling effect, as these factors were already higher at baseline in the high-risk group. The high-risk group, comprising more call center workers (47.0%), experiences constant monitoring and restricted autonomy, which may help explain the weaker associations of these factors with quality of life in this group. Taken together, these findings highlight the importance of considering the unique characteristics and needs of different emotional labor groups when designing and implementing interventions to promote psychological well-being in the workplace. Future research should further investigate the complex interplay between emotional labor, organizational factors, and individual coping mechanisms to develop more targeted and effective interventions for promoting psychological well-being among workers in various emotional labor contexts.

Meditation-induced changes in positive and negative affect have been well-documented in the literature [16,28]. A meta-analysis revealed an estimated effect size of 0.34 for the effect of meditation on negative emotions, and of 0.25 for its effect on positive emotions [16]. Moreover, a previous study reported that the use of smartphone-based mindfulness applications resulted in positive affect [70], suggesting that meditation can be used as a cost-eﬀective and convenient tool for improving affect. Consistent with these reports, we observed immediate changes in positive and negative affect after MBT. Prior research grounded in AET has demonstrated through longitudinal studies that employees’ momentary affective experiences at work influence the development of job attitudes, work behaviors, and employee well-being over time [25]. These findings highlight the importance of understanding affective trajectories through longitudinal assessment. Future intervention research could benefit from incorporating longitudinal designs with appropriate control groups to examine patterns of affective experiences over time. Such research on affective dynamics could inform the development of more targeted interventions for emotional labor workers.

Given the well-established effects of meditation on autonomic regulation [71,72], our exploratory physiological analysis focused on identifying which dimensions of emotional labor might relate to physiological responsiveness to MBT rather than demonstrating its physiological effects *per se*. Interestingly, the decrease in heart rate was positively correlated with baseline scores of *emotional demand and regulation*, suggesting that employees who exert greater effort to regulate their emotions and display diverse emotional expressions at work exhibited more pronounced physiological relaxation after MBT. One possible explanation is that these employees might experience greater stress relief due to higher baseline emotional strain. Alternatively, these employees may be more practiced in emotion regulation, which could facilitate their understanding and application of the techniques used in MBT. Both interpretations highlight the potential value of meditation-based interventions for employees in emotionally demanding roles. While the small sample limits group comparisons of physiological responsiveness, exploring which dimensions of emotional labor relate to physiological responses may provide potential insights for personalized workplace intervention design. Even within the same risk group, workers may vary in their responsiveness depending on their specific emotional labor characteristics or coping strategies, yet little is known about such moderating factors in emotional labor contexts. Future studies should examine whether specific dimensions such as emotional regulation predict responsiveness to meditation interventions, which could inform the design of more tailored programs and improve prediction of intervention outcomes.

Given the growing adoption of digital wellness programs in the workplace, the adaptability of the current MBT program to online formats warrants consideration. Our previous research demonstrated that a 4-week online MBT program incorporating the core components used in this study (stretching and mindfulness breathing) significantly reduced anxiety and trait anger among healthcare workers [30]. These findings suggest that the current MBT program could be effectively adapted to digital delivery formats. Online programs offer distinct advantages, including greater accessibility, scalability, and the potential to facilitate sustained practice over extended periods rather than single-session interventions. Considering that in-person delivery may include real-time expert guidance, enhanced participant engagement, and a stronger sense of organizational support, a hybrid approach combining initial in-person sessions with digital follow-up may optimize intervention effects.

This study has several limitations. While this study addressed emotional labor from both individual and organizational perspectives, employees’ coping strategies such as surface acting or deep acting were not assessed. Subtyping and profiling based on these coping strategies could potentially offer more insights for developing tailored interventions. Future research incorporating such behavioral measures or qualitative interviews could help elucidate the mechanisms underlying differences in risk groups. In addition, the present study did not assess participants’ subjective experiences with the MBT program. Future studies would benefit from incorporating qualitative measures, such as semi-structured interviews or open-ended survey questions, to explore how participants perceive the intervention, what barriers they encounter in applying the techniques to their daily work routines, and whether they find the program feasible and sustainable in real-world workplace settings. Such qualitative data would provide valuable insights for refining the intervention and enhancing its practical applicability. Second, the observational nature of this study and the absence of a control group limit the ability to attribute the observed changes solely to the MBT program. Other factors, including potential placebo effects, cannot be ruled out. In particular, for physiological measures such as resting heart rate, reductions observed after MBT may partly reflect natural adjustment to the environment rather than the program effect. Future studies with a control group of emotional laborers not receiving MBT intervention would provide stronger evidence for the effectiveness of the MBT intervention. Third, while the study included a large sample for psychological measures, the sample size for physiological measures was relatively small. This subset was selected based on participant availability, workplace scheduling, and limitations in equipment, resulting in a non-random, restricted sample. Consequently, the generalizability of findings on physiological changes and their links to emotional labor subscales is limited. Specifically, the null findings for heart rate variability warrant further investigation given the insufficient statistical power in the present study. Future research would benefit from larger sample sizes with a priori power analyses and randomized sampling procedures to better capture the physiological changes associated with workplace meditation interventions. Fourth, the study sample consisted predominantly of female participants (over 90%), which limits the generalizability of the findings to male emotional laborers. This gender imbalance is likely due to the nature of the occupations included in the study, such as customer service and call center work. Baseline differences between male and female are summarized in S1 Table. Although sensitivity analyses restricted to female employees yielded results consistent with the main findings (S2 and S3 Tables), the small proportion of male participants in this study, together with known gender differences in emotional labor [73], indicates that the applicability of the present findings to male employees is limited. Future studies should recruit more balanced samples to clarify potential gender-specific patterns in emotional labor and to examine whether the present findings can be applied to male workers. Lastly, the relatively low adjusted R² values observed in the regression models suggest that other unmeasured factors may have contributed to depressed mood in emotional labor workers. Future studies should consider incorporating additional psychological, interpersonal, and organizational variables, as well as objective measures, to better account for the variance in psychological outcomes. In addition, due to the lack of multiple comparison corrections, the possibility of false positive associations cannot be ruled out. The regression results should therefore be interpreted with caution. Nevertheless, the present study has the strength of identifying distinct psychological profiles among high-risk emotional laborers, which may provide groundwork data for future controlled studies aimed at investigating vulnerable groups.

## Conclusion

This study revealed important differences in psychological profiles and intervention responses between high-risk and low-risk emotional labor groups. *Emotional disharmony and hurt* was consistently associated with poorer psychological well-being across both groups, while *lack of a supportive and protective system in the organization* showed a stronger association with psychological difficulties in the high-risk group. Both groups demonstrated reduced heart rates and negative affect following the MBT, though the high-risk group showed significantly greater reductions in negative affect. These findings suggest that detailed consideration of workers’ emotional labor profiles encompassing both individual emotional experiences and organizational support factors should be incorporated when designing individualized workplace stress management programs.

## Supporting information

S1 Fig Flow chart of the present study. (DOCX)

S1 Table Baseline demographic, work-related, and outcome measures of male vs. female employees. SD, standard deviation; EL1, emotional demand and regulation; EL2, overload and conflict in customer service; EL3, emotional disharmony and hurt; EL4, organizational surveillance and monitoring; EL5, lack of a supportive and protective system in the organization. ^a, b^ indicates p values derived from independent samples t-tests and chi-square tests, respectively.
(DOCX)

S2 Table Stepwise multiple linear regression analysis examining associations between emotional labor subscales and psychological well-being among female employees. Data are presented as standardized beta coefficients (t value). ^**^ *p* < 0.001, ^*^ *p* < 0.05. EL1: emotional demand and regulation, EL2: overload and conflict in customer service, EL3: emotional disharmony and hurt, EL4: organizational surveillance and monitoring, EL5: lack of a supportive and protective system in the organization, QOL: quality of life.
(DOCX)

S3 Table Changes following the MBT among female employees. Data are given as adjusted mean (standard error). ^*^p < .05, ^**^p < .001.
(DOCX)

S4 Table Changes following the MBT by workplace type. Data are given as adjusted mean (standard error). ^*^p < .05, ^**^p < .001.
(DOCX)

## References

[1] Grandey AA. Emotion regulation in the workplace: a new way to conceptualize emotional labor. J Occup Health Psychol. 2000;5(1):95–110. doi: 10.1037//1076-8998.5.1.95 10658889

[2] Gross JJ. Antecedent- and response-focused emotion regulation: divergent consequences for experience, expression, and physiology. J Pers Soc Psychol. 1998;74(1):224–37. doi: 10.1037//0022-3514.74.1.224 9457784

[3] Jeung DY, Kim C, Chang SJ. Emotional labor and burnout: a review of the literature. Yonsei Med J. 2018;59(2):187–93. doi: 10.3349/ymj.2018.59.2.187 29436185 PMC5823819

[4] Mann S. ‘People-work’: emotion management, stress and coping. Br J Guid Couns. 2004;32(2):205–21. doi: 10.1080/0369880410001692247

[5] Zapf D. Emotion work and psychological well-being: a review of the literature and some conceptual considerations. Hum Resourc Manage Rev. 2002;12(2):237–68.

[6] Lee SR. A study on the current status, risk factors, and health effects of emotional labor among emotional labor workers. Ulsan: Korea Occupational Safety and Health Agency; 2015.

[7] Fouquereau E, Morin AJS, Lapointe É, Mokounkolo R, Gillet N. Emotional labour profiles: associations with key predictors and outcomes. Work Stress. 2018;33(3):268–94. doi: 10.1080/02678373.2018.1502835

[8] Nguyen N, Cheung F, Stinglhamber F. Emotional labor: a two-wave longitudinal person-centered approach. Int J Stress Manage. 2022;29(1):1–13. doi: 10.1037/str0000232

[9] Park SK, Heo HC, Sakong J, Jeon MJ. Emotional labor and job types of male firefighters in Daegu Metropolitan City. Ann Occup Environ Med. 2019;31:e25. doi: 10.35371/aoem.2019.31.e25 31620302 PMC6779900

[10] Demerouti E, Bakker AB, Nachreiner F, Schaufeli WB. The job demands-resources model of burnout. J Appl Psychol. 2001;86(3):499–512. doi: 10.1037/0021-9010.86.3.499 11419809

[11] Bakker AB, Demerouti E. Job demands-resources theory: taking stock and looking forward. J Occup Health Psychol. 2017;22(3):273–85. doi: 10.1037/ocp0000056 27732008

[12] Bakker AB, Demerouti E. The job demands‐resources model: state of the art. J Manager Psychol. 2007;22(3):309–28. doi: 10.1108/02683940710733115

[13] Azam MA, Katz J, Fashler SR, Changoor T, Azargive S, Ritvo P. Heart rate variability is enhanced in controls but not maladaptive perfectionists during brief mindfulness meditation following stress-induction: a stratified-randomized trial. Int J Psychophysiol. 2015;98(1):27–34. doi: 10.1016/j.ijpsycho.2015.06.005 26116778

[14] Krygier JR, Heathers JAJ, Shahrestani S, Abbott M, Gross JJ, Kemp AH. Mindfulness meditation, well-being, and heart rate variability: a preliminary investigation into the impact of intensive Vipassana meditation. Int J Psychophysiol. 2013;89(3):305–13. doi: 10.1016/j.ijpsycho.2013.06.017 23797150

[15] Ospina MB, Bond K, Karkhaneh M, Buscemi N, Dryden DM, Barnes V, et al. Clinical trials of meditation practices in health care: characteristics and quality. J Altern Complement Med. 2008;14(10):1199–213. doi: 10.1089/acm.2008.0307 19123875

[16] Sedlmeier P, Eberth J, Schwarz M, Zimmermann D, Haarig F, Jaeger S, et al. The psychological effects of meditation: a meta-analysis. Psychol Bull. 2012;138(6):1139–71. doi: 10.1037/a0028168 22582738

[17] Woods-Giscombe CL, Conklin J, Dodd A, Barthold LF, Perry Y, Brooks J, et al. Workplace meditation interventions for reducing psychological stress and other cardiovascular risk factors: workplace wellness policy implications. Curr Cardiovasc Risk Rep. 2022;16(12):231–40. doi: 10.1007/s12170-022-00708-9

[18] Pyri F, Abedi P, Maraghi E, Jefreh MG. The effect of mindfulness on quality of life among women with premature ovarian insufficiency: a randomized clinical trial. J Midlife Health. 2021;12(2):116–21. doi: 10.4103/jmh.JMH_66_20 34526745 PMC8409706

[19] Sadeghian B, Abedi P, Hamid N, Maraghi E, Molavi S. The effect of 8-week mindfulness counseling on sexual self-efficacy of women suffering from human immunodeficiency syndrome: a randomized controlled trial in Iran. Health Sci Rep. 2024;7(3):e1956. doi: 10.1002/hsr2.1956 38469109 PMC10926193

[20] Slagter HA, Davidson RJ, Lutz A. Mental training as a tool in the neuroscientific study of brain and cognitive plasticity. Front Hum Neurosci. 2011;5:17. doi: 10.3389/fnhum.2011.00017 21347275 PMC3039118

[21] Phongsuphap S, Pongsupap Y, Chandanamattha P, Lursinsap C. Changes in heart rate variability during concentration meditation. Int J Cardiol. 2008;130(3):481–4. doi: 10.1016/j.ijcard.2007.06.103 17764770

[22] Takahashi T, Murata T, Hamada T, Omori M, Kosaka H, Kikuchi M, et al. Changes in EEG and autonomic nervous activity during meditation and their association with personality traits. Int J Psychophysiol. 2005;55(2):199–207. doi: 10.1016/j.ijpsycho.2004.07.004 15649551

[23] Heckenberg RA, Eddy P, Kent S, Wright BJ. Do workplace-based mindfulness meditation programs improve physiological indices of stress? A systematic review and meta-analysis. J Psychosom Res. 2018;114:62–71. doi: 10.1016/j.jpsychores.2018.09.010 30314581

[24] Weiss HM, Cropanzano R. Affective events theory. Res Organ Behav. 1996;18(1):1–74.

[25] Wegge J, Dick R van, Fisher GK, West MA, Dawson JF. A test of basic assumptions of affective events theory (AET) in call centre work1. Br J Manage. 2006;17(3):237–54. doi: 10.1111/j.1467-8551.2006.00489.x

[26] Bakker AB, Demerouti E, Euwema MC. Job resources buffer the impact of job demands on burnout. J Occup Health Psychol. 2005;10(2):170–80. doi: 10.1037/1076-8998.10.2.170 15826226

[27] Hobfoll SE. The influence of culture, community, and the nested‐self in the stress process: advancing conservation of resources theory. Appl Psychol. 2001;50(3):337–421. doi: 10.1111/1464-0597.00062

[28] Jung Y-H, Ha TM, Oh CY, Lee US, Jang JH, Kim J, et al. The effects of an online mind-body training program on stress, coping strategies, emotional intelligence, resilience and psychological state. PLoS One. 2016;11(8):e0159841. doi: 10.1371/journal.pone.0159841 27479499 PMC4968838

[29] Lee D, Lee WJ, Choi S-H, Jang J-H, Kang D-H. Long-term beneficial effects of an online mind-body training program on stress and psychological outcomes in female healthcare providers: a non-randomized controlled study. Medicine (Baltimore). 2020;99(32):e21027. doi: 10.1097/MD.0000000000021027 32769863 PMC7593019

[30] Lee D, Kang DH, Ha NH, Oh CY, Lee U, Kang SW. Effects of an online mind–body training program on the default mode network: an EEG functional connectivity study. Sci Rep. 2018;8(1):1–8. 30446714 10.1038/s41598-018-34947-x PMC6240056

[31] Heppner WL, Shirk SD. Mindful moments: a review of brief, low‐intensity mindfulness meditation and induced mindful states. Soc Personal Psychol Compass. 2018;12(12). doi: 10.1111/spc3.12424

[32] Tang Y-Y, Hölzel BK, Posner MI. The neuroscience of mindfulness meditation. Nat Rev Neurosci. 2015;16(4):213–25. doi: 10.1038/nrn3916 25783612

[33] Tang Y-Y, Ma Y, Fan Y, Feng H, Wang J, Feng S, et al. Central and autonomic nervous system interaction is altered by short-term meditation. Proc Natl Acad Sci U S A. 2009;106(22):8865–70. doi: 10.1073/pnas.0904031106 19451642 PMC2690030

[34] Zeidan F, Johnson SK, Diamond BJ, David Z, Goolkasian P. Mindfulness meditation improves cognition: evidence of brief mental training. Conscious Cogn. 2010;19(2):597–605. doi: 10.1016/j.concog.2010.03.014 20363650

[35] Zeidan F, Johnson SK, Gordon NS, Goolkasian P. Effects of brief and sham mindfulness meditation on mood and cardiovascular variables. J Altern Complement Med. 2010;16(8):867–73. doi: 10.1089/acm.2009.0321 20666590

[36] Chang S, Kang H, Kim S, Kim I, Kim J, Kim H, et al. The development of Korean emotional labor scale and Korean violence scale. Seoul: The Korea Occupational Safety and Health Agency; 2014.

[37] Chang S. A study on the validation of Korean emotional labor scale (K-ELS) and Korean workplace violence scale (K-WVS). Korean occupational safety & health agency. 2014.

[38] Kim JI, Yun J-Y, Park H, Park S-Y, Ahn Y, Lee H, et al. A mobile videoconference-based intervention on stress reduction and resilience enhancement in employees: randomized controlled trial. J Med Internet Res. 2018;20(10):e10760. doi: 10.2196/10760 30348630 PMC6234345

[39] Choi S, Ko K, Park JB, Lee K-J, Lee S, Jeong I. Combined effect of emotional labor and job insecurity on sleep disturbance among customer service workers. Ann Occup Environ Med. 2020;32:e33. doi: 10.35371/aoem.2020.32.e33 33072344 PMC7533295

[40] Park H, Kim JI, Oh S, Kim J-H. The impact of emotional labor on the severity of PTSD symptoms in firefighters. Compr Psychiatry. 2018;83:53–8. doi: 10.1016/j.comppsych.2018.03.002 29573652

[41] Ryu H-Y, Hyun D-S, Jeung D-Y, Kim C-S, Chang S-J. Organizational climate effects on the relationship between emotional labor and turnover intention in Korean firefighters. Saf Health Work. 2020;11(4):479–84. doi: 10.1016/j.shaw.2020.08.007 33329914 PMC7728701

[42] Back C-Y, Hyun D-S, Jeung D-Y, Chang S-J. Mediating effects of burnout in the association between emotional labor and turnover intention in Korean clinical nurses. Saf Health Work. 2020;11(1):88–96. doi: 10.1016/j.shaw.2020.01.002 32206378 PMC7078559

[43] Kim JE, Park JH, Park SH. Anger suppression and rumination sequentially mediates the effect of emotional labor in Korean nurses. Int J Environ Res Public Health. 2019;16(5):799. doi: 10.3390/ijerph16050799 30841533 PMC6427706

[44] Hyun DS, Jeung DY, Kim C, Ryu HY, Chang SJ. Does emotional labor increase the risk of suicidal ideation among firefighters? Yonsei Med J. 2020;61(2):179–85. doi: 10.3349/ymj.2020.61.2.179 31997627 PMC6992451

[45] Kroenke K, Spitzer RL, Williams JBW, Löwe B. The patient health questionnaire somatic, anxiety, and depressive symptom scales: a systematic review. Gen Hosp Psychiatry. 2010;32(4):345–59. doi: 10.1016/j.genhosppsych.2010.03.006 20633738

[46] Arey DL, Gerbi A, Sagi A. A randomized controlled trial on single-session mindfulness self-compassion interventions for Fibromyalgia Syndrome: evaluating interoceptive awareness, anxiety, and pain. Curr Psychol. 2024;43(47):36234–45. doi: 10.1007/s12144-024-07049-3

[47] Müller C, Dubiel D, Kremeti E, Lieb M, Streicher E, Siakir Oglou N, et al. Effects of a single physical or mindfulness intervention on mood, attention, and executive functions: results from two randomized controlled studies in university classes. Mindfulness. 2021;12(5):1282–93. doi: 10.1007/s12671-021-01601-z

[48] Palmer R, Roos C, Vafaie N, Kober H. The effect of ten versus twenty minutes of mindfulness meditation on state mindfulness and affect. Sci Rep. 2023;13(1):20646. doi: 10.1038/s41598-023-46578-y 38001316 PMC10673854

[49] Sleimen-Malkoun R, Devillers-Réolon L, Temprado J-J. A single session of mindfulness meditation may acutely enhance cognitive performance regardless of meditation experience. PLoS One. 2023;18(3):e0282188. doi: 10.1371/journal.pone.0282188 36920902 PMC10016675

[50] Jang JH, Kim J-H, Yun J-Y, Choi S-H, An SC, Kang D-H. Differences in functional connectivity of the insula between brain wave vibration in meditators and non-meditators. Mindfulness. 2018;9:1857–66. 30524515 10.1007/s12671-018-0928-x PMC6244630

[51] Jang JH, Jung WH, Kang D-H, Byun MS, Kwon SJ, Choi C-H, et al. Increased default mode network connectivity associated with meditation. Neurosci Lett. 2011;487(3):358–62. doi: 10.1016/j.neulet.2010.10.056 21034792

[52] Hopper SI, Murray SL, Ferrara LR, Singleton JK. Effectiveness of diaphragmatic breathing for reducing physiological and psychological stress in adults: a quantitative systematic review. JBI Database Syst Rev Implement Rep. 2019;17(9):1855–76. doi: 10.11124/JBISRIR-2017-003848 31436595

[53] Allen M, Dietz M, Blair KS, van Beek M, Rees G, Vestergaard-Poulsen P, et al. Cognitive-affective neural plasticity following active-controlled mindfulness intervention. J Neurosci. 2012;32(44):15601–10. doi: 10.1523/JNEUROSCI.2957-12.2012 23115195 PMC4569704

[54] Taren AA, Gianaros PJ, Greco CM, Lindsay EK, Fairgrieve A, Brown KW, et al. Mindfulness meditation training and executive control network resting state functional connectivity: a randomized controlled trial. Psychosom Med. 2017;79(6):674–83. doi: 10.1097/PSY.0000000000000466 28323668 PMC5489372

[55] Quaglia JT, Zeidan F, Grossenbacher PG, Freeman SP, Braun SE, Martelli A, et al. Brief mindfulness training enhances cognitive control in socioemotional contexts: behavioral and neural evidence. PLoS One. 2019;14(7):e0219862. doi: 10.1371/journal.pone.0219862 31323050 PMC6641506

[56] Garland E, Gaylord S, Park J. The role of mindfulness in positive reappraisal. Explore (NY). 2009;5(1):37–44. doi: 10.1016/j.explore.2008.10.001 19114262 PMC2719560

[57] Lim Y-J, Yu B-H, Kim D-K, Kim J-H. The positive and negative affect schedule: psychometric properties of the korean version. Psychiatry Investig. 2010;7(3):163–9. doi: 10.4306/pi.2010.7.3.163 20927304 PMC2947803

[58] Noh S, Kaspar V, Xinyin Chen. Measuring depression in Korean immigrants: assessing validity of the translated Korean version of CES-D scale. Cross-Cult Res. 1998;32(4):358–77. doi: 10.1177/106939719803200403

[59] Min SK, Lee CI, Kim KI, Suh SY, Kim DK. Development of Korean version of WHO quality of life scale abbreviated version (WHOQOL-BREF). J Korean Neuropsychiatric Assoc. 2000;39(3):571–9.

[60] Lumma A-L, Kok BE, Singer T. Is meditation always relaxing? Investigating heart rate, heart rate variability, experienced effort and likeability during training of three types of meditation. Int J Psychophysiol. 2015;97(1):38–45. doi: 10.1016/j.ijpsycho.2015.04.017 25937346

[61] Shaffer F, Ginsberg JP. An overview of heart rate variability metrics and norms. Front Public Health. 2017;5:258. doi: 10.3389/fpubh.2017.00258 29034226 PMC5624990

[62] Billman GE. The LF/HF ratio does not accurately measure cardiac sympatho-vagal balance. Frontiers Media SA. 2013;:26. 10.3389/fphys.2013.00026 PMC3576706 23431279

[63] Kim GH, Lee HS, Jung SW, Lee JG, Lee JH, Lee KJ, et al. Emotional labor, workplace violence, and depressive symptoms in female Bank employees: a questionnaire survey using the K-ELS and K-WVS. Ann Occup Environ Med. 2018;30:17. doi: 10.1186/s40557-018-0229-9 29564140 PMC5848577

[64] Lee J, Han C, Ko Y-H, Kang J, Byun Y, Son Y, et al. Emotional changes and protective factors of emotional workers in the public and private sector. Psychiatry Investig. 2020;17(7):645–53. doi: 10.30773/pi.2019.0329 32571004 PMC7385212

[65] Lawrence SA, Troth AC, Jordan PJ, Collins AL. A review of emotion regulation and development of a framework for emotion regulation in the workplace. The role of individual differences in occupational stress and well being. 2011;9:197–263.

[66] Vonderlin R, Biermann M, Bohus M, Lyssenko L. Mindfulness-based programs in the workplace: a meta-analysis of randomized controlled trials. Mindfulness. 2020;11(7):1579–98. doi: 10.1007/s12671-020-01328-3

[67] Errazuriz A, Schmidt K, Undurraga EA, Medeiros S, Baudrand R, Cussen D, et al. Effects of mindfulness-based stress reduction on psychological distress in health workers: a three-arm parallel randomized controlled trial. J Psychiatr Res. 2022;145:284–93. doi: 10.1016/j.jpsychires.2020.11.011 33199052

[68] Wharton AS. The affective consequences of service work: Managing emotions on the job. Work Occup. 1993;20(2):205–32.

[69] Ashforth BE, Humphrey RH. Emotional labor in service roles: the influence of identity. Acad Manage Rev. 1993;18(1):88–115.

[70] Wen L, Sweeney TE, Welton L, Trockel M, Katznelson L. Encouraging mindfulness in medical house staff via Smartphone App: a pilot study. Acad Psychiatry. 2017;41(5):646–50. doi: 10.1007/s40596-017-0768-3 28795335

[71] Solberg EE, Ekeberg O, Holen A, Ingjer F, Sandvik L, Standal PA, et al. Hemodynamic changes during long meditation. Appl Psychophysiol Biofeedback. 2004;29(3):213–21. doi: 10.1023/b:apbi.0000039059.20738.39 15497620

[72] Tyagi A, Cohen M. Yoga and heart rate variability: a comprehensive review of the literature. Int J Yoga. 2016;9(2):97–113. doi: 10.4103/0973-6131.183712 27512317 PMC4959333

[73] Yang S-B, Guy ME. Gender effects on emotional labor in Seoul metropolitan area. Public Personnel Manage. 2014;44(1):3–24. doi: 10.1177/0091026014550491

