# Supplementary figures and images for: Distinct psychological profiles and responsiveness to a brief intervention in workers with high versus low intensity emotional labor: an observational study
Source: PLoS One. 2026 May 6;21(5):e0345553. doi: 10.1371/journal.pone.0345553 (PMC13148714; doi:10.1371/journal.pone.0345553)

**Figure S1.** Flow chart of the present study.


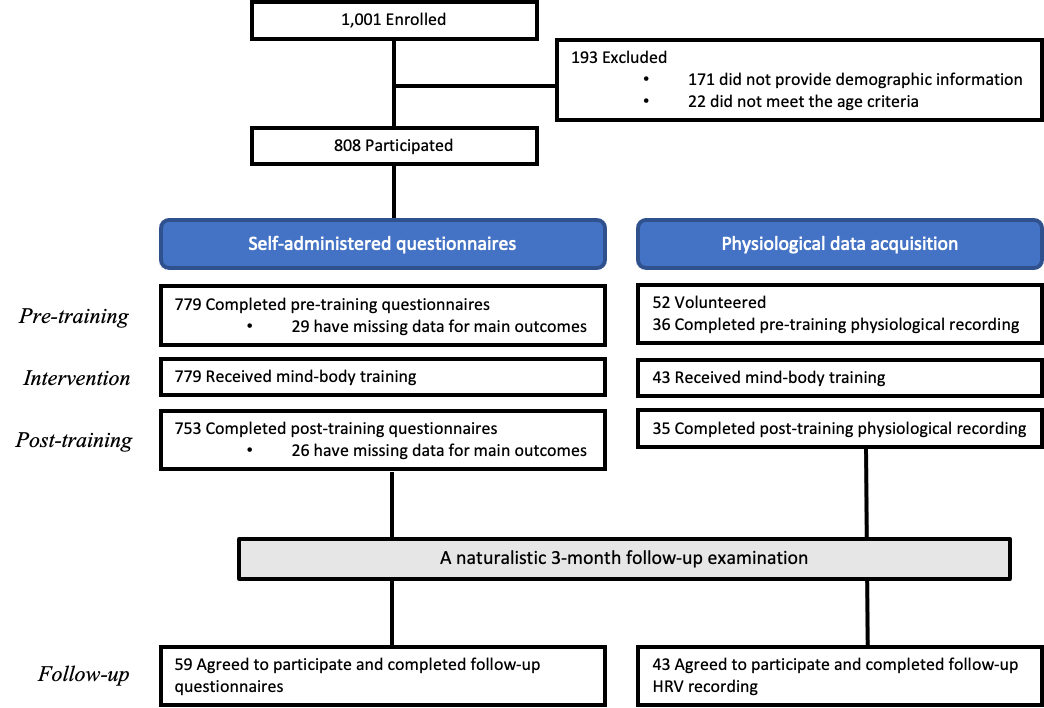

Supplement: S1 Fig — (DOCX) [file pone.0345553.s001.docx]
