# Supplementary material for: Distinct psychological profiles and responsiveness to a brief intervention in workers with high versus low intensity emotional labor: an observational study
Source: PLoS One. 2026 May 6;21(5):e0345553. doi: 10.1371/journal.pone.0345553 (PMC13148714; doi:10.1371/journal.pone.0345553)
Supplement: S1 Table — SD, standard deviation; EL1, emotional demand and regulation; EL2, overload and conflict in customer service; EL3, emotional disharmony and hurt; EL4, organizational surveillance and monitoring; EL5, lack of a supportive and protective system in the organization. a, b indicates p values derived from independent samples t-tests and chi-square tests, respectively. (DOCX) [file pone.0345553.s002.docx]

**Table** **S1. Baseline demographic, work-related, and outcome measures of male vs. female employees**

| **Variable** | | **Group** | | | **Statistics** | |
| --- | --- | --- | --- | --- | --- | --- |
|  | | Male (*n* = 71) | Female (*n* = 652) |  | |  |
|  | | N (%) or mean (SD) | N (%) or mean (SD) |  | | *p* |
| **Demographic features** | |  |  |  | |  |
| Age, year | | 33.31 (8.28) | 36.80 (8.37) |  | | **.001**^a^ |
| Married, % | | 24 (33.8) | 362 (55.5) |  | | **.041**^b^ |
| University degree, % | | 51 (71.8) | 502 (77.0) |  | | .571^b^ |
| **Work-related features** | |  |  |  | |  |
| Length of employment, months | | 63.74 (84.41) | 68.74 (75.03) |  | | .603^a^ |
| Permanent employment, % | | 47 (66.2) | 414 (63.5) |  | | .584^b^ |
| Weekly working hours | | 41.82 (5.26) | 42.15 (5.96) |  | | .676^a^ |
| Workplace, % | |  |  |  | | **<.001**^b^ |
| Hospital | | 29 (40.8) | 231 (35.4) |  | |  |
| Civil affairs centers | | 5 (7.0) | 174 (26.7) |  | |  |
| Call centers | | 37 (52.1) | 247 (37.9) |  | |  |
| Emotional labor score | |  |  |  | |  |
| EL1 | | 70.70 (16.77) | 73.16 (15.67) |  | | .213^a^ |
| EL2 | | 59.04 (23.79) | 65.24 (23.53) |  | | **.040**^a^ |
| EL3 | | 42.46 (20.59) | 57.81 (21.00) |  | | **<.001**^a^ |
| EL4 | | 37.56 (17.79) | 47.14 (22.54) |  | | **.001**^a^ |
| EL5 | | 45.08 (15.79) | 49.57 (14.34) |  | | **.013^a^** |
| **Psychological measures** |  | |  |  | |  |
| Positive affect | 25.17 (6.63) | | 21.49 (7.02) |  | | **<.001**^a^ |
| Negative affect | 18.15 (6.52) | | 18.81 (7.27) |  | | .470^a^ |
| Depressed mood | 15.10 (8.68) | | 17.65 (8.90) |  | | **.027**^a^ |
| Quality of life |  | |  |  | |  |
| physical | 14.16 (2.22) | | 13.35 (2.33) |  | | **.006**^a^ |
| psychological | 12.96 (2.91) | | 11.86 (2.54) |  | | **.001**^a^ |
| social | 13.00 (2.51) | | 13.16 (2.42) |  | | .605^a^ |
| environment | 12.92 (2.80) | | 12.12 (2.34) |  | | **.008**^a^ |
