# Supplementary material for: Distinct psychological profiles and responsiveness to a brief intervention in workers with high versus low intensity emotional labor: an observational study
Source: PLoS One. 2026 May 6;21(5):e0345553. doi: 10.1371/journal.pone.0345553 (PMC13148714; doi:10.1371/journal.pone.0345553)
Supplement: S2 Table — Data are presented as standardized beta coefficients (t value). ** p < 0.001, * p < 0.05. EL1: emotional demand and regulation, EL2: overload and conflict in customer service, EL3: emotional disharmony and hurt, EL4: organizational surveillance and monitoring, EL5: lack of a supportive and protective system in the organization, QOL: quality of life. (DOCX) [file pone.0345553.s003.docx]

**Table** **S2.** Stepwise multiple linear regression analysis examining associations between emotional labor subscales and psychological well-being among female employees

|  | **High-risk group (*n* = 305)** | | | | |  | |
| --- | --- | --- | --- | --- | --- | --- | --- |
|  | Independent variables | | | | | Statistics | |
| Dependent variables | EL1 | EL2 | EL3 | EL4 | EL5 | *R*^2^ | *F* |
| Depressed mood | 0.024 (0.43) | -0.05 (-0.89) | **0.32 (5.70**)** | -0.09 (-1.50) | **0.14 (2.48*)** | .131 | **22.71**** |
| QOL, Physical | -0.09 (-1.55) | -0.01 (-0.08) | **-0.24 (-4.29**)** | 0.01 (0.12) | 0.05 (0.88) | .055 | **18.39**** |
| QOL, Psychological | -0.01 (-0.15) | 0.07 (1.12) | **-0.18 (-3.21**)** | 0.10 (1.70) | -0.01 (-0.12) | .030 | **10.32*** |
| QOL, Social | 0.01 (0.13) | 0.04 (0.61) | **-0.19 (-3.35**)** | 0.08 (1.35) | -0.04 (-0.64) | .033 | **11.22*** |
| QOL, Environmental | 0.04 (0.60) | 0.06 (0.99) | **-0.22 (-3.96**)** | 0.02 (0.29) | -0.09 (-1.57) | .046 | **15.68**** |
|  | **Low-risk group (*n* = 347)** | | | | |  | |
|  | Independent variables | | | | | Statistics | |
| Dependent variables | EL1 | EL2 | EL3 | EL4 | EL5 | *R*^2^ | *F* |
| Depressed mood | 0.02 (0.37) | -0.02 (-0.29) | **0.23 (4.23**)** | 0.03 (0.49) | 0.01 (0.25) | .049 | **17.90**** |
| QOL, Physical | -0.01 (-0.15) | -0.07 (-1.25) | **-0.24 (-4.46**)** | **-0.12 (-2.14*)** | 0.02 (0.32) | .083 | **16.46**** |
| QOL, Psychological | 0.06 (1.11) | **-0.16 (-3.02*)** | **-0.24 (-4.44**)** | -0.07 (-1.14) | 0.02 (0.45) | .102 | **20.29**** |
| QOL, Social | **0.13 (2.33*)** | **-0.16 (-2.86*)** | **-0.20 (-3.59**)** | -0.03 (-0.46) | -0.02 (-0.39) | .078 | **10.47**** |
| QOL, Environmental | **0.12 (2.41^*^)** | -0.09 (-1.57) | **-0.27 (-4.98**)** | **-0.14 (-2.30*)** | -0.03 (-0.60) | .106 | **14.49**** |
