# Supplementary material for: Distinct psychological profiles and responsiveness to a brief intervention in workers with high versus low intensity emotional labor: an observational study
Source: PLoS One. 2026 May 6;21(5):e0345553. doi: 10.1371/journal.pone.0345553 (PMC13148714; doi:10.1371/journal.pone.0345553)
Supplement: S3 Table — Data are given as adjusted mean (standard error). *p < .05, **p < .001. (DOCX) [file pone.0345553.s004.docx]

**Table** **S3.** Changes following the MBT among female employees

| **Self-reported questionnaire** | | | | | | | |
| --- | --- | --- | --- | --- | --- | --- | --- |
|  | **High-risk (*n* = 305)** | | **Low-risk (*n* = 347)** | | **F** | | |
|  | **Pre** | **Post** | **Pre** | **Post** | **Time** | **Group** | **Interaction** |
| **Positive affect** | 20.69 (0.40) | 22.20 (0.38) | 27.97 (0.45) | 29.03 (0.42) | **308.48^**^** | **6.42^*^** | 0.56 |
| **Negative affect** | 20.32 (0.41) | 13.25 (0.26) | 17.48 (0.38) | 12.14 (0.25) | **324.76^**^** | **24.15^**^** | **11.88^*^** |
