# Supplementary material for: Distinct psychological profiles and responsiveness to a brief intervention in workers with high versus low intensity emotional labor: an observational study
Source: PLoS One. 2026 May 6;21(5):e0345553. doi: 10.1371/journal.pone.0345553 (PMC13148714; doi:10.1371/journal.pone.0345553)
Supplement: S4 Table — Data are given as adjusted mean (standard error). *p < .05, **p < .001. (DOCX) [file pone.0345553.s005.docx]

**Table** **S4.** Changes following the MBT by workplace type

| **Self-reported questionnaire** | | | | | | | | | |
| --- | --- | --- | --- | --- | --- | --- | --- | --- | --- |
|  | **Hospitals (*n* = 260)** | | **Civil affairs centers (*n* = 179)** | | **Call centers (n = 284)** | | **F** | | |
|  | **Pre** | **Post** | **Pre** | **Post** | **Pre** | **Post** | **Time** | **Group** | **Interaction** |
| **Positive affect** | 21.69 (0.45) | 25.34 (0.48) | 22.22 (0.53) | 30.89 (0.57) | 21.77 (0.43) | 26.70 (0.46) | **355.71^**^** | **7.35^*^** | **16.75**^**^ |
| **Negative affect** | 19.06 (0.45) | 12.29 (0.29) | 17.85 (0.54) | 11.56 (0.35) | 19.01 (0.43) | 13.84 (0.28) | **321.75^**^** | **5.97^*^** | **4.18^*^** |
